# Neutrophil Leukocyte: Combustive Microbicidal Action and Chemiluminescence

**DOI:** 10.1155/2015/794072

**Published:** 2015-12-13

**Authors:** Robert C. Allen

**Affiliations:** Department of Pathology, Creighton University School of Medicine, Omaha, NE 68131, USA

## Abstract

Neutrophil leukocytes protect against a varied and complex array of microbes by providing microbicidal action that is simple, potent, and focused. Neutrophils provide such action via redox reactions that change the frontier orbitals of oxygen (O_2_) facilitating combustion. The spin conservation rules define the symmetry barrier that prevents direct reaction of diradical O_2_ with nonradical molecules, explaining why combustion is not spontaneous. In burning, the spin barrier is overcome when energy causes homolytic bond cleavage producing radicals capable of reacting with diradical O_2_ to yield oxygenated radical products that further participate in reactive propagation. Neutrophil mediated combustion is by a different pathway. Changing the spin quantum state of O_2_ removes the symmetry restriction to reaction. Electronically excited singlet molecular oxygen (^1^O_2_^*^) is a potent electrophilic reactant with a finite lifetime that restricts its radius of reactivity and focuses combustive action on the target microbe. The resulting exergonic dioxygenation reactions produce electronically excited carbonyls that relax by light emission, that is, chemiluminescence. This overview of neutrophil combustive microbicidal action takes the perspectives of spin conservation and bosonic-fermionic frontier orbital considerations. The necessary principles of particle physics and quantum mechanics are developed and integrated into a fundamental explanation of neutrophil microbicidal metabolism.

## 1. Introduction

Considered as an organ, the collective mass of hematopoietic bone marrow in a healthy adult is greater than that of the liver. The major proportion of this hematopoietic activity is directed to producing neutrophil leukocytes. Each day a healthy human adult releases about a hundred billion neutrophils into the circulating blood [1]. This baseline production is greatly expanded in inflammatory states and by treatment with a granulocyte-colony stimulating factor (G-CSF) such as filgrastim [2]. In addition to stimulating neutrophil hyperplasia, that is, increased production, G-CSF treatment also results in hypertrophic changes, that is, larger neutrophils with severalfold greater azurophilic granule content and proportionally increased myeloperoxidase (MPO) [3].

The neutrophil serves as the principal leukocyte of the acute inflammatory response and is the primary microbicidal phagocyte of the innate and acquired immune defense systems. Accomplishing such function starts in the circulation with intricate neutrophil-endothelial contact. When stimulated by microbial products, complement activation peptides, cytokines,* et cetera*, neutrophils fuse their specific (a.k.a., secondary) granules with their cytoplasmic membrane (i.e., specific degranulation), thus providing the increased surface-to-volume ratio necessary for locomotion and exposing the cytokine receptors and opsonin receptors required for close endothelial contact and diapedesis (i.e., transit) from the vascular space through the endothelial lining into the tissue interstitial space. Chemotactic locomotion to the site of infection is directed by concentration gradients of bacterial products, anaphylatoxin, cytokines,* et cetera*.

Contact and receptor-mediated recognition of an opsonified (e.g., complement-, immunoglobulin-labeled) microbe results in phagocytosis, formation of a phagosome, and fusion with azurophilic (a.k.a. primary) granules containing myeloperoxidase to produce the phagolysosome [4].

## 2. Respiratory Burst Metabolism

The morphologic changes of phagocytosis are associated with magnitudinal increase in hexose monophosphate shunt metabolism of glucose and with proportionally increased molecular oxygen (O_2_) consumption, that is, the respiratory burst. This mitochondria-independent metabolic activity is required for effective microbicidal action [5, 6]. The resulting microbicidal oxygenation reactions have exergonicities sufficient to produce electronic excited products yielding light emission in the visible spectrum, that is, chemiluminescence or luminescence [7].

Phagocytosis is linked to activation of NADPH oxidase. This complex flavocytochrome oxidase drives the respiratory burst [6, 8]. Two reducing equivalents (i.e., two electrons (e^−^) plus two protons (H^+^)) are transferred from NADPH to the oxidase where they distribute in a manner allowing for univalent (one equivalent) reduction of O_2_, yielding hydrodioxylic acid (HO_2_; a.k.a. hydroperoxyl radical) [9, 10]. HO_2_ is an acid with a p*K*_a_ of 4.9 and, as such, dissociates yielding a proton and its conjugate base, the superoxide anion (O_2_^−^). Production of HO_2_ within the neutrophil phagosome or phagolysosome dynamically acidifies the confined space. As the pH of the space approaches the p*K*_a_ (i.e., 4.9), the ratio of HO_2_ to O_2_^−^ approaches unity, removing the anionic barrier to direct radical-radical disproportionation [10, 11]. In such acidic milieu, HO_2_ reacts directly with O_2_^−^ to produce hydrogen peroxide (H_2_O_2_) and singlet molecular oxygen (^1^O_2_^*∗*^) [10–12].

**Figure 1:**
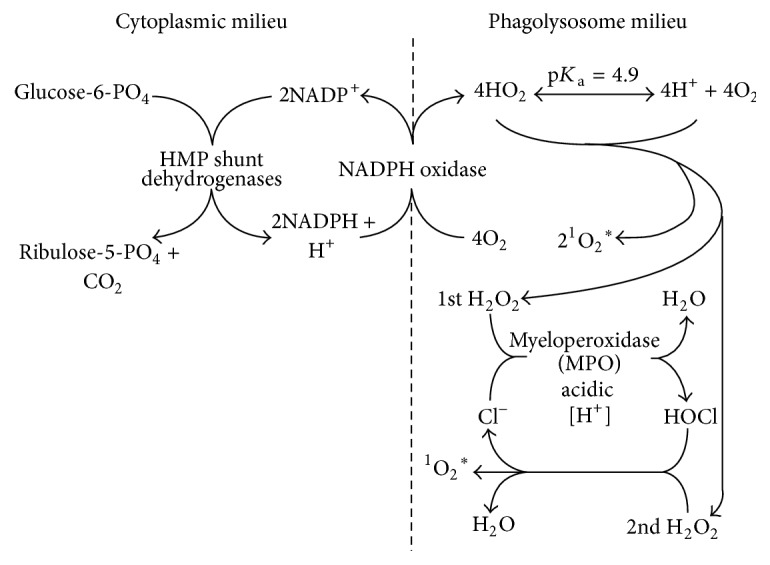
Schematic depiction of neutrophil HOCl and ^1^O_2_^*∗*^ generation.

NADPH oxidase dynamically acidifies the phagolysosome and produces H_2_O_2_. These activities provide the optimal milieu and H_2_O_2_ substrate for myeloperoxidase (MPO) oxidation of chloride (Cl^−^) to the chloronium (Cl^+^) state yielding hypochlorous acid (HOCl) [13]. Spontaneous reaction of HOCl with an additional H_2_O_2_ yields Cl^−^, H_2_O, and ^1^O_2_^*∗*^ [14]. Both HOCl and ^1^O_2_^*∗*^ are potent microbicidal reactants. The metabolic generation of these reactants is depicted in Figure 1.

## 3. Oxygen Reactivity

With regard to electronegativity, oxygen is second only to fluorine. The order of electronegativity values for fluorine (F), oxygen (O), and chorine (Cl) is 4.0, 3.5, and 3.0, respectively [15]. The energy difference separating O_2_ from water (H_2_O) is large, that is, a difference of 1.23 volts (V) or 96.5 kilocalories per mole (kcal mol^−1^), and provides the driving force of life on earth.

The Merriam-Webster Dictionary defines combustion as a chemical reaction that occurs when oxygen combines with other substances to produce heat and usually light. As such, neutrophil microbicidal action is combustive. Dioxygenation reactions are among the most exergonic in biology. The heat of reaction calculations for the reaction of O_2_ with ethylene liberates 93 kcal mol^−1^ (i.e., 389 kilojoules per mole (kJ mol^−1^)) or the energy equivalent to an einstein (i.e., one mole) of ultraviolet photons [16]. The electronegativity of oxygen predicts the high exergonicity of oxygenation reactions, but such reactions are not spontaneous. Placed together in a chamber, O_2_ does not react with ethylene. For reaction to occur, a photon or spark with energy sufficient to initiate ethylene bond homolytic cleavage must be applied.

This presentation addresses the questions: why does not oxygen spontaneously react with organic material? Why is oxygen reduction, a process that decreases its thermodynamic potential, required as a first step for neutrophil combustive microbicidal action? And what is the best viewpoint for considering oxygen reactivity? The review provides the background physics and chemistry for a fundamental perspective with regard to oxygen reactivity in particular, neutrophil combustive microbicidal action with its associated luminescence, and reaction chemistry in general. Hopefully, it will encourage interested researchers out of their comfort zone and broaden their scientific outlook.

## 4. Particle Physics, Quantum Mechanics, and Reaction Symmetry

Particles are of two symmetry types, each with unique statistical mechanical properties [17, 18]. They are either Bose-Einstein particles (bosons) or Fermi-Dirac particles (fermions). According to the exchange principle, a pair of particles, that is,* a* and* b*, can be described by a wave function, Ψ(*a*, *b*), representing the space and spin coordinates of the particles. Exchanging the particles generates a new wave function, Ψ(*b*, *a*). Even if the particles are indistinguishable (e.g., electrons), the particle sites are distinguishable. Each site is unique with regard to its spin-state, that is, spin-up (↑) or spin-down (↓). Each combination is distinct. For indistinguishable particles, the result of exchange can differ by no more than a quantum phase factor. There are only two symmetry possibilities. Exchange can be symmetric: Ψ(*a*, *b*) = Ψ(*b*, *a*); or exchange can be antisymmetric: Ψ(*a*, *b*) = −Ψ(*b*, *a*) [19].

### 4.1. Bosons

The wave functions of bosons are symmetric to exchange of a pair of particles; that is, Ψ(*a*, *b*) = Ψ(*b*, *a*). Bosons obey ordinary commutation; *a* × *b* = *b* × *a*. Rotating a boson through 360 degrees, Ψ—360° → Ψ, returns it to its original state. Bosons are symmetric particles with integral spin. Photons, the force carrier particles of electromagnetic energy, are bosons and are described by the Planck equation: *E* = *hν*, where *E* is energy, *h* is Planck's constant, and *ν* is frequency.

Two antisymmetric fermions can also couple to produce a symmetric bosonic product. Such products include large bosons with mass, such as alpha (*α*) particles. The character of atomic and molecular orbitals can likewise be considered as bosonic or fermionic [20]. As will be developed subsequently, the frontier orbital of an atom or molecule is bosonic if composed of paired antisymmetric electrons. As such, the highest fully occupied atomic or molecular orbital (HO(A)MO) has bosonic character. Such atoms and molecules are nonradical and diamagnetic.

### 4.2. Fermions

The wave functions of fermions are antisymmetric to exchange of particles; that is, Ψ(*a*, *b*) = −Ψ(*b*, *a*). Spin is an intrinsic property of the particle and is quantized. Fermions are characterized as having half-integer spin and appear as multiples of the basic unit (1/2)*ħ*, where *ħ* (*h*-bar) equals Planck's constant (*h*) divided by 2*π*. Fermions anticommute; that is, *a* × *b* ≠ *b* × *a*. Rotating a fermion through 360 degrees, Ψ—360° → −Ψ, changes the phase but does not return the fermion to its original state. An additional 360 degrees' rotation, −Ψ—360° → Ψ, is required to return the antisymmetric particle to its original state.

Fermions compose the solid stuff of the universe and are subject to time. Electrons, protons, and neutrons are fermions. The frontier orbitals of atoms, such as hydrogen (H), nitrogen (N), and oxygen (O), and molecules, such as O_2_ and nitrous oxide (NO), have singly occupied atomic or molecular orbitals (SO(A)MO). An orbital with a single electron has fermionic character. Atoms and molecules with fermionic orbitals are radical and paramagnetic.

### 4.3. Principal, Radial, and Angular Quantum Numbers

Atomic hydrogen (H) is composed of a positively charged nuclear proton (H^+^) and a negatively charged electron (e^−^). Both H^+^ and e^−^ are fermions. H^+^ is thousandfold more massive than e^−^, and, as such, the kinetic and potential energies of e^−^ are described as its “orbit” relative to the massive H^+^. The orbital wave function describes the energy possibilities. Using polar coordinates, the position of e^−^ can be described as its distance,* r*, from its nucleus and two angles, *θ* and *φ*. The total wave function is isolated into 3 separate contributions, Ψ(*r*, *θ*, *φ*) = *R*(*r*)Θ(*θ*)Φ(*φ*), where *R*(*r*) is the radial component and Θ(*θ*) and Φ(*φ*) are the angular components. Solution of each component yields a quantum number. The radial component yields the principal quantum number, *n*. The angular components yield the azimuthal quantum number, *l*, and the magnetic quantum number, *m*_*l*_.

The principal quantum number,* n*, describes the energy of the orbital. The degree of orbital degeneracy is the square of the principal quantum number,* n* ^2^. When *n* = 1, the degeneracy is 1^2^ = 1, yielding the 1s orbital. When *n* = 2, the degeneracy is 2^2^ = 4, yielding the 2s, 2p_*x*_, 2p_*y*_, and 2p_*z*_ orbitals. The azimuthal quantum number,* l*, describes the shape of the orbit and the orbital angular momentum of e^−^. The magnetic quantum number, *m*_*l*_, describes the number of orbitals with a given value of *l*. The value of the total orbital angular momentum,* L*, is *L* = √[*l*(*l* + 1)]*ħ*.

### 4.4. Spin Quantum Number

Electrons and other fermions possess intrinsic angular momentum that is independent of orbital motion. This intrinsic quantum mechanical property, that is, spin, is described by the spin quantum number,* s*. The magnitude of *s* is restricted to a value of 1/2. The total spin angular momentum,* S*, of a system is expressed by the equation *S* = √[*s*(*s* + 1)]*ħ*. The intrinsic spin with its value of (1/2)*ħ* (abbreviated to 1/2) is a quality of fermions without analogy in classical physics. Just as *l* gives rise to *m*_*l*_,* s* gives rise to the spin quantum number *m*_*s*_. Only two values are allowed for *m*_*s*_. When *m*_*s*_ = 1/2, e^−^ is described as spin-up (↑); when *m*_*s*_ = −1/2, e^−^ is described as spin-down (↓).

### 4.5. Pauli Exclusion Principle

**Figure 2:**
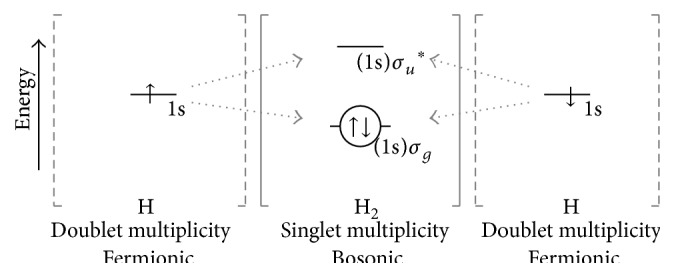
Combination of two doublet multiplicity hydrogen atoms (H, shown on the left and right of the graphic) yields singlet multiplicity molecular hydrogen (H_2_, shown in the center of the graphic). Multiplicity is defined as |2(*S*)| + 1, where *S* is the total spin number. Constructive overlap of the two antisymmetric 1s SOAOs of the H atoms yields the HOMO *σ* of H_2_. The (1s) notation before *σ* indicates the atomic source of the molecular orbital. *σ* ^*∗*^ is the LUMO antibonding orbital (*∗* indicates antibonding) of H_2_. The energy differences in the figures are for illustration and not drawn to scale.

Each e^−^ of an atom or molecule is defined by its five quantum numbers: *n*, *l*, *m*_*l*_, *s*, and *m*_*s*_. The Pauli exclusion principle states that no two electrons of a given atom can have identical quantum numbers; that is, the total wave function for a system must be antisymmetric to the exchange of any pair of electrons. For an orbit to accommodate two electrons, the electrons must have opposite spins. If one orbital e^−^ has *m*_*s*_ = 1/2  (↑), the other orbital e^−^ must have *m*_*s*_ = −1/2  (↓). As such, the total spin quantum number, *S*, for an orbital electron-couple is 1/2 + −1/2 = 0  (↑ ↓). Orbital coupling of the fermionic electrons results in spin-neutralization. In effect, the antisymmetric fermions combine into a symmetric boson. This concept is illustrated in Figure 2 by the reaction of two H atoms to generate molecular H_2_.

### 4.6. Multiplicity

Multiplicity is defined as |2(*S*)|+1, where *S* is the total spin number. Multiplicity is a spectroscopic term and indicates the number of wave functions possible for the system; that is, singlet indicates 1, doublet indicates 2, triplet indicates 3,* et cetera*. In its ground (lowest energy) electronic state, atomic H has a single e^−^ in the 1s orbital and has an *S* value of 1/2 or −1/2; thus |2(1/2  or  −1/2)| + 1 = 2; the multiplicity is doublet. For molecular H_2_ the value of *S* is 0; thus, |2(0)| + 1 = 1; the multiplicity is singlet. In Figure 2, the orbital possibilities are depicted by a horizontal bar (—). The spin quantum number of each e^−^ is represented as spin-up (*m*_*s*_ = 1/2 = ↑) or spin-down (*m*_*s*_ = −1/2 = ↓). The circle surrounding the *σ* orbital electron-couple of the HOMO of H_2_ symbolizes the bosonic character of the filled orbital.

Orbital overlap of the two H atoms can be constructive or destructive. When the electrons are antisymmetric, that is, ↑ and ↓, overlap is constructive resulting in chemical bonding; that is, there is an increased probability of finding electrons in the internuclear region between the two H nuclei. Combining the two atomic 1s orbitals yields the bonding sigma molecular orbital, *σ*. Note that bonding lowers the energy of the system. When the electrons are ↑ and ↑ or ↓ and ↓, the overlap is destructive and no bonding occurs.

## 5. Frontier Orbital Theory

Chemical reaction involves frontier orbital interaction. Frontier orbital theory focuses on the initial orbital conditions of the reactants and on reactive transition with special emphasis on the highest occupied and lowest unoccupied orbitals [21, 22]. The frontier orbitals, that is, the highest occupied atomic or molecular orbital (HO(A)MO) and the lowest unoccupied molecular orbital (LUMO), define the reactive possibilities. As depicted in Figure 2, the electrons of the singly occupied atomic orbital (SOAO) of the two H atoms constructively overlap in a radical-radical (i.e., doublet-doublet) annihilation producing the *σ* bonding HOMO orbital of singlet multiplicity, diamagnetic molecular hydrogen (H_2_).

For the H atoms, the electrons of the 1s orbitals have identical energies, but with covalent bonding to form H_2_, the wave functions overlap and split producing two molecular orbitals, one with lower and one with higher energy than the original 1s atomic orbitals. The bonding orbital (*σ*) is lower in energy and stable, thus promoting bonding. The antibonding orbital (*σ* ^*∗*^) is of higher energy. Populating the antibonding orbital promotes bond breaking [23]. Consequently, if a photon of sufficient frequency is captured by a *σ* electron, its electronic excitation to the *σ* ^*∗*^ orbital results in H_2_ bond cleavage.

## 6. Wigner-Witmer Spin Conservation and Boson-Fermion Orbital Symmetry

The Wigner-Witmer spin conservation rules describe reaction symmetry possibilities in terms of reactant and product multiplicities [24, 25]. The spin conservation rules state that the overall spin angular momentum of a system must be conserved. The symmetries of reactants and products must correlate. Reactant and product state possibilities can also be considered in terms of fermionic and bosonic frontier orbital character [20].

In Table 1 reactants and products are presented in terms of multiplicity and also in terms of bosonic or fermionic orbital character. As depicted in Figure 2, combining two doublet multiplicity H atoms produces singlet multiplicity H_2_. With regard to orbital spin character, the two antisymmetric fermionic electrons of the atomic orbitals are coupled (condensed or combined) resulting in the bosonic electron pair of the *σ* orbital of H_2_. Since the Pauli principle requires the total wave function for any system of electrons to be antisymmetric to exchange an orbital pair of electrons, only phase-opposite electrons can occupy a given orbital state. Orbital coupling of such fermionic electrons imposes bosonic character.

### 6.1. Hund's Maximum Multiplicity Rule

**Figure 3:**
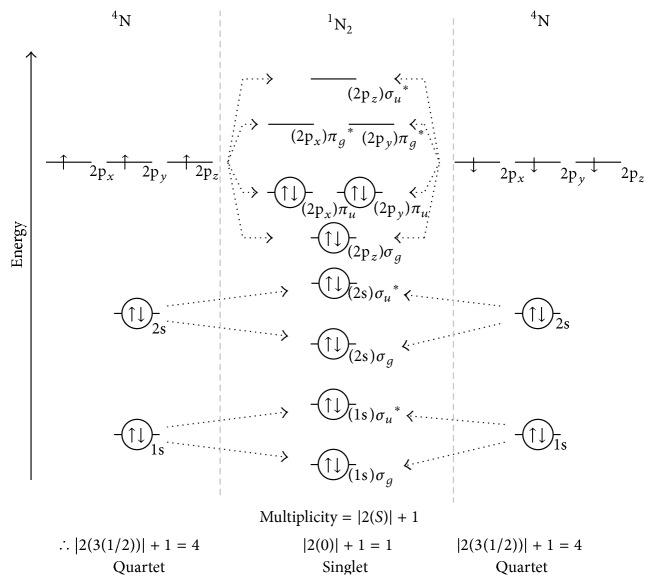
Combination of two quartet multiplicity nitrogen atoms (^4^N; the superscript 4 indicates multiplicity) to yield singlet multiplicity molecular nitrogen (^1^N_2_).

Hund's maximum multiplicity rule states that the electronic configuration with highest multiplicity has the lowest energy. The greater the number of wave functions possible for a system, the lower the energy.  Figure 3 depicts the combination of two nitrogen atoms (N) to yield molecular nitrogen (N_2_). Ground state atomic N is a paramagnetic triradical with one electron in each of its three 2p orbitals. Note that each electron has the same *m*_*s*_ value resulting in quartet multiplicity; that is, |2(3(1/2  or  −1/2))|+1 = 4. The orbitals of higher multiplicity states are more contracted than those of lower multiplicity. Higher multiplicity states produce greater nuclear-electron attraction and are of lower energy [26].

Combining the atomic orbitals of the two quartet multiplicity nitrogen atoms (N) generates the filled (bosonic) (1s and 2s) *σ* and *σ* ^*∗*^ orbitals; constructive overlap of the phase-opposite electrons of the three 2p orbitals of the two N atoms generates one *σ* and two *π* bonds of triple-bonded ground state singlet multiplicity N_2_. The triradical N atoms combine to produce nonradical N_2_. From the fermion-boson frontier orbital perspective, each N presents a complex of trifermionic SOAOs, that is, Ψ (i.e., *S* = 3(1/2)) or −Ψ (i.e., *S* = 3(−1/2)). Consistent with Table 1, antisymmetric coupling produces triple-bonded bosonic (i.e., *S* = 0) N_2_.

### 6.2. Oxygen Chemistry

**Figure 4:**
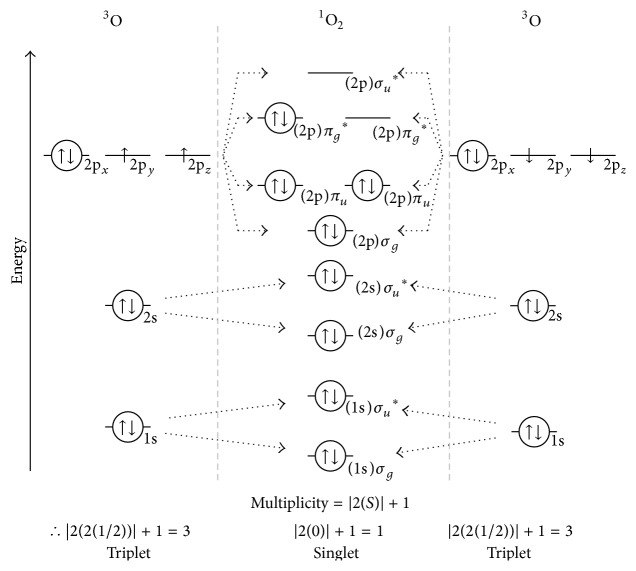
Combination of two triplet multiplicity oxygen atoms (^3^O) to yield electronically excited singlet multiplicity molecular hydrogen (^1^O_2_^*∗*^).

Frontier orbital overlap of two oxygen atoms with antisymmetric SOMO is constructive producing ^1^O_2_^*∗*^ as depicted in Figure 4. As per Table 1, combining two ground state triplet multiplicity paramagnetic, diradical oxygen atoms (O) produces an electronically excited singlet multiplicity, diamagnetic, nonradical ^1^O_2_^*∗*^. Note the product ^1^O_2_^*∗*^ obeys the spin symmetry rules but violates Hund's maximum multiplicity rule. As such, ^1^O_2_^*∗*^ is electronically excited (indicated by *∗*) with an energy of 22.5 kcal mol^−1^ greater than that of triplet multiplicity ground state O_2_ [14].

**Figure 5:**
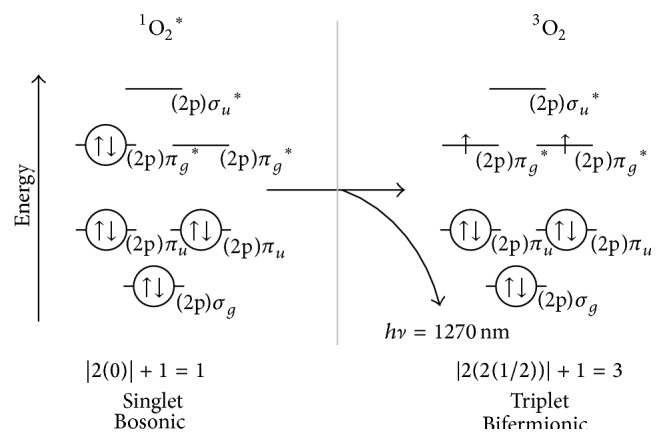
Relaxation of singlet molecular oxygen to its triplet ground state by infrared photon emission. For simplicity, only the (2p) *σ*, *π*, *π* ^*∗*^, and *σ* ^*∗*^ orbitals of O_2_ are shown.

The spin conservation rules predict that the change in spin multiplicity, that is, a singlet-to-triplet transition, is of low probability. As such, electronically excited ^1^O_2_^*∗*^ is metastable with a relatively long half-life. The estimated four-microsecond lifetime of ^1^O_2_^*∗*^ is sufficient to allow its participation as an electrophilic reactant in dioxygenation reactions. However, such reactions are restricted to within a radius of about 0.2 microns (*μ*m) from its point of generation [27, 28]. As depicted in Figure 5, unreacted ^1^O_2_^*∗*^ relaxes to its triplet multiplicity ground state (^3^O_2_) by emitting a 1270 nm (near infrared) photon.

As per the maximum multiplicity rule, ground state molecular oxygen is a triplet multiplicity, paramagnetic diradical with one e^−^ occupying each of its two *π* ^*∗*^ SOMOs; it is bifermionic. Ground state O_2_ can be in either the Ψ (i.e., *S* = 1/2 + 1/2 = 2(1/2) = 1) or the −Ψ (i.e., *S* = −1/2 − 1/2 = 2(−1/2) = −1) state; thus, the multiplicity is |2(1) + 1| = 3, that is, triplet.

The vast majority of biological molecules are singlet multiplicity—that is, *S* = 0—and, as such, present bosonic frontier orbitals. For such molecules, chemistry is confined to bosonic HOMO-LUMO exchange of a composite orbital electron-couple. The fermionic orbital character of radicals is atypical and favors radicals-radical reaction. Antisymmetric fermionic frontier orbital overlap is constructive resulting in covalent bonding of the bosonic singlet product. As described in Table 1, constructive SOMO-SOMO overlap of a doublet reactant and a triplet reactant produces a doublet product; that is, the fermionic character is preserved.

Direct reaction of ground state triplet multiplicity O_2_ with singlet multiplicity organic molecules violates the spin conservation rules, and, as such, combustion is not spontaneous. Overlap of the bifermionic *π* ^*∗*^ (the 2 SOMO) frontier orbitals of triplet multiplicity O_2_ with the empty LUMO or bosonic HOMO of singlet multiplicity organic molecules is not constructive. Such reactions are symmetry-restricted, and the only product possible would also be a bifermionic triplet. O_2_ with its triplet multiplicity ground state is relatively exotic. The triplet multiplicity states of most molecules are electronically excited. The relaxation of such excited triplet multiplicity molecules to their singlet ground state, that is, triplet-to-singlet transition, violates the conservation rules and is of low probability. The delayed relaxation of an excited triplet to its singlet ground state by photon emission is responsible for the phenomenon of phosphorescence [29].

### 6.3. Neutrophils Change the Spin Number of O_2_

Considering the complexity and variety of potentially pathogenic organisms, neutrophil microbicidal action must be simple, potent, and focused so as to maximize microbicidal action and minimize collateral damage. Microbicidal combustion is realized by changing the frontier orbitals of oxygen from bifermionic to bosonic. Neutrophil combustive action is expected to produce light. Oxygenation reactions yield electronically excited products that relax to ground state by emission of light in the visible spectral range. Bioluminescence and chemiluminescence reactions are essentially limited to such oxygenation reactions. Neutrophil luminescence is energetic proof of such combustive activity.

Respiratory burst metabolism mobilizes the reducing equivalents required for changing the frontier orbitals of O_2_. One equivalent (radical) reduction is unusual in cytoplasmic metabolism. When it does occur, the semiquinone of a riboflavin prosthetic group is typically involved. Such flavoenzymes mark the departure from two equivalent cytoplasmic transfers to one equivalent cytochrome transfer. One equivalent reduction of O_2_ changes its multiplicity from triplet to doublet, thus reducing its overall fermionic orbital character. The flavocytochrome enzyme NADPH oxidase catalyzes the reduction of bifermionic triplet multiplicity O_2_ to fermionic doublet multiplicity HO_2_ [9, 10]. Acidic dissociation of HO_2_ yields its conjugate base, doublet multiplicity superoxide anion (O_2_^−^) [11]. As per Figure 1 and Table 1, direct SOMO-SOMO orbital overlap of HO_2_ with O_2_^−^ produces singlet multiplicity ground state H_2_O_2_ plus ^1^O_2_^*∗*^ [10, 12].

The H_2_O_2_ produced serves as substrate for myeloperoxidase (MPO) oxidation of chloride producing hypochlorous acid; that is, H_2_O_2_ + Cl^−^→ H_2_O + HOCl. Note that all reactants and products are exclusively singlet multiplicity (bosonic). The hypochlorous acid (HOCl) produced reacts directly with additional H_2_O_2_. This reaction involves the bosonic HOMO of H_2_O_2_ and the bosonic LUMO of HOCl producing the intermediate singlet multiplicity chloroperoxy acid (HOOCl) and singlet multiplicity H_2_O [14]. Disintegration of the chloroperoxy intermediate yields ground state singlet multiplicity chloride and electronically excited singlet multiplicity oxygen. The net reaction is H_2_O_2_ + HOCl →^1^O_2_^*∗*^ + H_2_O + Cl^−^ + H^+^. The bosonic orbital symmetry of ^1^O_2_^*∗*^ allows direct constructive spin-allowed overlap with the bosonic frontier orbitals of biological substrates producing singlet multiplicity dioxygenated products of bosonic symmetry.

In conventional combustive burning, sufficient energy must be applied to a nonradical (singlet) substrate to produce homolytic bond cleavage yielding two radical (doublet) products. The fermionic SOMO of these doublet multiplicity radical products can now constructively overlap with one of the fermionic SOMOs of triplet multiplicity ground state O_2_. As described in Table 1, the product of doublet-triplet reaction will have doublet multiplicity. Such radical-diradical reactions yield heat plus additional radical products that can further participate in the radical propagation process of burning.

Neutrophil microbicidal action is combustion by a different pathway. Instead of radicalizing a substrate to facilitate reaction with diradical O_2_, the bifermionic orbitals of ground state triplet O_2_ are converted to the bosonic orbitals of ^1^O_2_^*∗*^. In its electronically excited singlet multiplicity state, the bosonic frontier orbitals of oxygen can participate in spin-allowed electrophilic oxygenation reactions with a broad spectrum of singlet multiplicity biological molecules. As per spin conservation, the products of these reactions are singlet multiplicity. Such products are nonradical, diamagnetic, and unable to participate in the radical propagation reactions that are characteristic of burning.

The potent electrophilic reactivity of ^1^O_2_^*∗*^ is restricted to its metastable lifetime. The microsecond lifetime range of ^1^O_2_^*∗*^ confines reactions to within about a 0.2 *μ*m radius from its point of origin [27, 28]. Thus, phagolysosomal generation of ^1^O_2_^*∗*^ in close proximity to the target microbe guarantees that combustive action is focused on the microbe with minimal collateral damage to the host. By changing the spin quantum number of oxygen, the neutrophil not only realizes its potent electrophilic microbicidal action, but also limits such combustive action to the target microbe.

### 6.4. Neutrophil Luminescence

**Figure 6:**
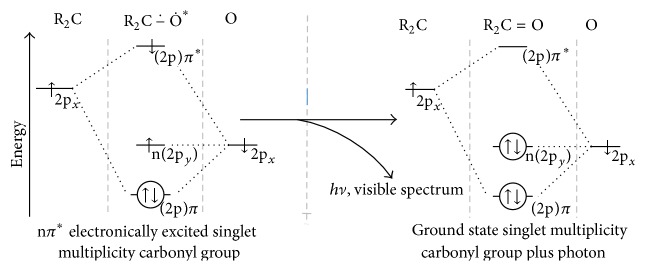
Relaxation (pi antibonding to nonbonding (*π* ^*∗*^ → n) electronic orbital transition) of a singlet electronically excited carbonyl product of dioxygenation to its singlet ground state by photon emission.

The endoperoxide and dioxetane products of dioxygenation are of relatively high energy. These dioxygenated products are typically unstable and of singlet multiplicity. Disintegration of such products yields two carbonyl functions. One carbonyl is in the ground singlet multiplicity state, and the other carbonyl is in the electronically excited n*π* ^*∗*^ singlet multiplicity state. As depicted in Figure 6, the n*π* ^*∗*^ description indicates that an electron from the ground state nonbonding (n) orbital of the atomic oxygen (O) component occupies the *π* ^*∗*^ orbital of the electronically excited carbonyl function. When the spin of the excited *π* ^*∗*^ electron is antisymmetric to that of the ground state n electron of O, the electronically excited carbonyl is singlet multiplicity. Thus, *π* ^*∗*^-to-n relaxation to ground state is spin-allowed and yields a photon with energy proportional to the difference between the two orbital states.

In bioluminescence and chemiluminescence processes, dioxygenated products disintegrate by oxygen-oxygen bond cleavage producing the singlet multiplicity n*π* ^*∗*^ electronically excited carbonyl. This electronically excited state is chemically generated. In fluorescence, electronic excitation to the n*π* ^*∗*^ state can occur when a photon of proper energy is captured by an electron in the nonbonding (n) orbital of the O component of the carbonyl.

As depicted in Figure 6, relaxation of the electron from *π* ^*∗*^ of the singlet excited carbonyl back to the n orbital of O results in photon (*hν*) emission and returns the carbonyl function to its ground singlet state. The frequency of the emitted photon is the energy difference that separates the n orbital from the *π* ^*∗*^ orbital. Photons are symmetric bosons; their absorbance or emission does not affect the electron spin.

In fluorescence, photon capture promotes an electron of an orbital to an appropriately higher energy orbital. Such photon excitation results in transient antibonding and fermionic character, but the overall spin symmetry is retained; that is, singlet character is retained. Relaxation of an n*π* ^*∗*^ electronically excited singlet multiplicity carbonyl to its singlet ground state by fluorescence or chemiluminescence is spin-allowed, and, as such, the lifetime of the excited state is very short-lived, typically in the nanosecond range. Consequently the excited carbonyl is unable to participate in reaction chemistry.

### 6.5. Native and Substrate Specific Chemiluminescence

The chemiluminescence produced by neutrophils is an energy byproduct of microbicidal combustion and varies with the molecular composition of the microbe. Different microbes present substrates that vary with regard to dioxygenation susceptibility and combustion quantum efficiencies, that is, the photon yield per dioxygenation reaction. Native luminescence is easily measured using a luminometer, but the photon yield is insufficient for measurement of less than a hundred thousand neutrophils. In order to increase photon yield and impose reaction specificity, chemiluminigenic substrates can be introduced to the milieu. Chemiluminigenic substrates are susceptible to dioxygenation producing endoperoxides or dioxetanes ultimately yielding n*π* ^*∗*^ electronically excited carbonyl products. Such substrates increase the sensitivity for detecting dioxygenation activity by several orders of magnitude [30]. In addition to increasing sensitivity, chemiluminigenic substrates can be selected for specificity with regard to the type of oxygenation activity measured [31].

The state of the circulating neutrophil reflects the state of inflammatory immune defense [32]. The sensitivity and specificity obtained using selective chemiluminigenic probe in combination with phagocytic and chemical stimuli allow simultaneous quantification of multiple neutrophil metrics using a microliter or less of unseparated whole blood. When subjected to discriminant function analysis, such neutrophil luminescence measurements allow assessment of host inflammatory state and hematopoietic marrow stress [32–35].

## 7. Neutrophil Myeloperoxidase and Lactic Acid Bacteria (LAB)

Neutrophil leukocytes respond to cytokines and other agents by fusing specific granules with the cytoplasmic membrane, that is, specific degranulation. Such fusion effectively increases the surface-to-volume ratio of the neutrophil, as is required for locomotion and phagocytosis, and for upregulated membrane expression of cytokine and opsonin receptors, as is required for effective recognition and phagocytosis. Microbicidal effectiveness is optimized by coordinating phagocytosis with activation of NADPH oxidase, resulting in dynamic acidification and H_2_O_2_ production. Fusion of the lysosomal azurophilic granules with the microbe-containing phagosome produces a phagolysosome with a relatively high MPO concentration in an acidic milieu replete with H_2_O_2_. The phagolysosomal milieu is optimal for combustive microbicidal action.

The neutrophils of patients with chronic granulomatous disease (CGD) have defective NADPH oxidase function and consequently have defective microbicidal combustion and do not chemiluminesce [36]. These CGD neutrophils phagocytose microbes and release MPO into the phagolysosomal space, but the neutrophil respiratory burst is defective and microbicidal action is poor. Consequently, CGD patients have an increased susceptibility to infection. Interestingly, the incidence of pneumococcal and other streptococcal infections is not increased in CGD patients; that is, infection rates for these microbes are about equivalent to those for healthy subjects [37]. Pneumococci and streptococci are cytochrome-deficient lactic acid bacteria (LAB) that produce lactic acid and H_2_O_2_ as metabolic products [38, 39]. Phagocytosis of live LAB by CGD neutrophils does result in microbicidal action. In the case of CGD neutrophils, the phagocytosed LAB provides the acid and H_2_O_2_ necessary for effective MPO-mediated microbicidal combustion and chemiluminescence [36].

Human neutrophils contain a relatively high concentration of MPO (about 5% of the dry weight of the neutrophil) in their azurophilic granules [40]. Following departure of the neutrophil leukocyte from the hematopoietic marrow and a relatively short transit time in the circulating blood, the neutrophil leaves the circulating and enters the tissue pool [1]. Neutrophils characteristically migrate to sites of injury and infection and accumulate in high concentrations. However, even in the absence of inflammation and infection, healthy humans have significant concentrations of neutrophils present in tissue fluids. The oral and vaginal cavities have neutrophil concentrations in proportion to blood neutrophil concentrations [41, 42].

When neutrophils transit from the blood to tissue and body spaces, they carry MPO into these spaces. Each neutrophil carries about four femtograms of MPO. As such, neutrophils deliver relatively large quantities of MPO into body spaces on a routine basis. The oral and vaginal cavities have acidic pH. The normal florae of these spaces are cytochrome-negative LAB that generate lactic acid and H_2_O_2_ as metabolic products. The transit of neutrophils into such spaces delivers MPO into milieux that are rich in LAB.

MPO selectively binds to Gram-negative and many Gram-positive bacteria. However, LAB show relatively poor MPO binding [43]. When small concentrations of neutrophil-free MPO are added to bacterial suspensions containing viridans streptococci with other competing bacteria, a potent synergistic microbicidal action is directed against MPO-binding Gram-positive, for example,* Staphylococcus aureus*, and Gram-negative, for example,* Pseudomonas aeruginosa* and* Escherichia coli*, microbes [44]. Damage to the LAB is only observed at significantly higher MPO concentrations. This LAB-MPO synergistic action may explain the dominance of streptococci and lactobacilli in healthy oral and vaginal cavities [45].

Selective binding of MPO to a microbe guarantees that ^1^O_2_^*∗*^ is generated in close proximity to the target microbe, resulting in selective, focused combustive microbicidal action. The ^1^O_2_^*∗*^ lifetime of a few microseconds limits damage to within about a 0.2 *μ*m radius of its point of generation [27, 28]. Thus, combustion is focused on potential pathogens with minimal collateral damage to the host cells and the normal LAB flora [44].

## 8. Conclusions and Perspectives

There are no restrictions on biologic evolution other than those imposed by physical reality. The spin conservation rules describe fundamental symmetry restrictions that define reactive possibilities. These rules forbid the direct reaction of triplet oxygen with singlet organic molecules, and, as such, combustion is not spontaneous. Chemical reactions involve frontier orbitals. Changing the multiplicity of oxygen changes its frontier orbitals, eliminating the symmetry barrier to its electrophilic reactive potential.

Neutrophils kill microbes by a unique, focused combustive action. The redox reactions of neutrophil respiratory burst metabolism change triplet ground state (diradical) O_2_ to singlet electronically excited (nonradical) ^1^O_2_^*∗*^. In addition, other singlet multiplicity (nonradical) microbicidal reactants, for example, H_2_O_2_ and HOCl, are generated. These agents are uniformly singlet multiplicity (nonradical) reactants with bosonic frontier orbitals. Their reactions with microbes are consistent with the spin conservation rules. ^1^O_2_^*∗*^ has one empty (LUMO) *π* ^*∗*^ orbital and one full (HOMO) *π* ^*∗*^ orbital with bosonic character. This highly electrophilic LUMO can participate in spin-allowed bosonic LUMO-HOMO reactions with biological molecules. The dioxygenated products of such combustions yield the electronically excited carbonyl functions that relax by photon emission, that is, luminescence or chemiluminescence.

Considering frontier orbital reactivity in the bosonic-fermionic terms of particle physics broadens the perspective for explaining oxygen chemistry in particular and for appreciating reaction chemistry in general. Biological reactions typically involve constructive frontier HOMO-LUMO overlap. Radical reactions involving singly occupied atomic or molecular orbitals (SO(A)MO) are uncommon in biology under normal conditions, that is, in the absence of high energy radiation or heat producing homolytic bond cleavage. Biological reactions involving one electron transfer are typically restricted to flavoenzymes and to highly ordered cytochrome electron transport systems such as those found in mitochondria. Cytoplasmic redox reactions typically involve two equivalent transfers and are nonradical. Orbital pairing of antisymmetric fermionic electrons imposes a bosonic (nonradical) character.

Chemical reactions can be generalized as constructive bosonic transfers (i.e., HOMO-LUMO) or constructive fermionic annihilations. Both reactions produce bosonic products. Radical-radical (i.e., SO(A)MO-SO(A)MO) orbital overlap is typically constructive; that is, fermionic radicals react with fermionic radicals. Overlap of a bosonic HOMO with a fermionic SO(A)MO is not constructive. As illustrated in Figure 1, the first steps of the hexose monophosphate metabolic pathway involve dehydrogenation of glucose-6-phosphate and reduction of NADP^+^ yielding NADPH; these redox reactions involve balanced two equivalent bosonic transfers. Bosonic orbital pair transfers are the norm in biochemistry. The bosonic nature of the antisymmetric orbital electron-couple may be essential for such biologic redox transfer. Any fermionic electron transfer would open the possibility for reaction with bifermionic oxygen.

There are advantages to transferring electrons as a bosonic orbital-couple. As stated by Dirac, “If a state has zero total angular momentum, the dynamical system is equally likely to have any orientation, and hence spherical symmetry occurs” [19]. A bosonic orbital electron-couple has *S* = 0. This has significance with regard to the Heisenberg uncertainty principle which states that the uncertainty of momentum (Δ*p*) multiplied by the uncertainty of position (Δ*x*) is always equal to or greater than (1/2)*ħ*; that is, Δ*p*Δ*x* ≥ (1/2)*ħ* [46]. The spin momentum of the bosonic orbital electron-couple is known, that is, zero. Consequently, the positional uncertainty of the electron-couple must be proportionally large. Thus, the intermolecular redox transfer of a bosonic orbital electron-couple may proceed by a quantum tunneling mechanism analogous to the emission of a bosonic alpha particle from an atomic nucleus in alpha radiation decay [20]. Such a quantum tunneling mechanism explains the facility for electron-couple transfer in biologic redox reactions.

## Figures and Tables

**Table 1:** Spin conservation rules. Multiplicity states from the perspective of the bosonic-fermionic orbital character of reactants and products.

Reactants	Product(s)
Singlet + singlet	Singlet
*Bosonic* + *bosonic*	*Bosonic*

Singlet + doublet	Doublet
*Bosonic* + *fermionic*	*Fermionic*

Singlet + triplet	Triplet
*Bosonic* + *bifermionic*	*Bifermionic*

Doublet + doublet	Singlet (triplet)^*∗*^
*Fermionic* + *fermionic*	*Bosonic (bifermionic)* ^*∗*^

Doublet + triplet	Doublet (quartet)^*∗*^
*Fermionic* + *bifermionic*	*Fermionic* (*trifermionic*)^*∗*^

Doublet + quartet	Triplet
*Fermionic* + *trifermionic*	*Bifermionic*

Triplet + triplet	Singlet
*Bifermionic* + *bifermionic*	*Bosonic*

Triplet + quartet	Doublet
*Bifermionic* + * trifermionic*	*Fermionic*

Quartet + quartet	Singlet
*Trifermionic* + * trifermionic*	*Bosonic*

^*∗*^The products in parentheses are symmetrically possible but improbable.
